# 2148 Understanding the health effects of binding and tucking for gender affirmation

**DOI:** 10.1017/cts.2018.268

**Published:** 2018-11-21

**Authors:** Tonia Poteat, Mannat Malik, Erin Cooney

**Affiliations:** Johns Hopkins Bloomberg School of Public Health

## Abstract

OBJECTIVES/SPECIFIC AIMS: Gender affirmation is a critical aspect of the health and well-being of transgender individuals. For many transgender people, this includes changing one’s physical appearance to align with one’s felt gender. Some gender-affirming body modifications require medical interventions such as hormone therapies and surgeries. Other modifications, such as tucking to create a flat-appearing lower pelvis and binding to create a flat-appearing chest, require no external intervention. The published literature is slowly growing on the health effects of gender affirming medical interventions; however, other body modifications are understudied. As part of our needs assessment of the transgender community, we sought to understand the frequency and health impact of binding and tucking. METHODS/STUDY POPULATION: A quantitative online survey was developed based on qualitative interviews with 20 community-based key informants. The survey was available online, in English, for 6 months. Eligible participants were 18 years of age or older, lived in the Baltimore metropolitan area, and identified as transgender and/or a sex different from what was assigned on their original birth certificate. RESULTS/ANTICIPATED RESULTS: 139 participants provided complete data: 45% were assigned male at birth (AMAB) and 55% were assigned female at birth (AFAB). In total, 54% were Black, 40% White, and 9% Latinx. Of AFAB participants, 80% had bound their chest tissue. Of those who had bound, 51% bound 7 days/week, 62% bound 8+ hours per day, and 68% were concerned about the health effects of binding. The most common symptoms associated with binding were back pain (65%), shortness of breath (48.6%), bad posture (32%), chest pain (30%), and light-headedness (30%). Of AMAB participants, 71% had ever tucked, 85% of those tucked 7 days per week, 79% tucked 8+ hours per day, and 50% were concerned about the health effects of tucking. Most common symptoms included itching (28%), rash (21%), testicular pain (17%), penile pain (14%), and skin infections (12%). DISCUSSION/SIGNIFICANCE OF IMPACT: The majority of transgender participants used binding or tucking for gender-affirming body modification and at least half of them have concerns about associated health effects. Clinicians should ask transgender patients about binding and tucking behaviors and assess for common symptoms. More research is needed to better understand the benefits and risks of gender-affirming binding and tucking behaviors.

